# Analysis of pregnancy-associated factors after fertility-sparing therapy in young women with early stage endometrial cancer or atypical endometrial hyperplasia

**DOI:** 10.1186/s12958-021-00808-y

**Published:** 2021-08-03

**Authors:** Yuan Fan, Xingchen Li, Jiaqi Wang, Yiqin Wang, Li Tian, Jianliu Wang

**Affiliations:** 1 Department of Obstetrics and Gynecology, Peking University People’s Hospital, Beijing, 100044 China; 2 Reproductive Medical Center, Peking University People’s Hospital, Beijing, 100044 China

**Keywords:** Fertility-sparing therapy, Conservative treatment, Endometrial cancer, Atypical endometrial hyperplasia, Assisted reproductive technology

## Abstract

**Background:**

Fertility-sparing therapy is an alternative conservative treatment for patients with early stage endometrioid cancer or atypical endometrial hyperplasia. In this study, we investigated pregnancy outcomes and pregnancy-associated factors in young patients receiving hormonal therapy.

**Methods:**

We retrospectively analyzed 68 patients who attempted to conceive after fertility-sparing therapy and achieving complete remission (CR). They were divided into a pregnancy group and a non-pregnancy group. A Cox proportional hazard regression model was applied for univariate and multivariate analysis to determine factors associated with pregnancy. Kaplan–Meier analysis, combined with the log-rank test, was used to calculate a patient’s pregnancy probability and the distribution of recurrence-free survival (RFS).

**Results:**

A total of 36 patients became pregnant with 47 pregnancies. Univariate and multivariate Cox analysis revealed that several factors were associated with pregnancy, including BMI at the time of pregnancy permission, the time to CR, prolonged treatment time, the number of hysteroscopy procedures, the endometrium thickness after CR, and relapse before pregnancy. The mean RFS of patients who achieved pregnancy, and those who did not, was 27.6 months and 14.8 months, respectively (*P* = 0.002). No significant difference was detected in terms of cumulative RFS when compared between assisted reproductive technology (ART) cases and those involving natural conception (NC) (*P* = 0.707).

**Conclusions:**

Normal BMI, a shorter time to CR, a prolonged three-month treatment, fewer hysteroscopy procedures, and a thicker endometrium may be positive indicators for successful pregnancies, while relapse before pregnancy may have a negative effect on conception. Moreover, a successful pregnancy protects the endometrium while ART does not increase the risk of recurrence.

## Background

Endometrial cancer (EC) is one of the most common gynecological malignancies worldwide. Approximately 7% of EC cases occur in women aged younger than 45 years; data indicate that the incidence of this disease is gradually increasing [1]. Atypical endometrial hyperplasia (AEH) is a precancerous lesion and 29% of such cases progress to EC within a few years [2]. As many as 70% of premenopausal patients with EC are nulliparous; consequently, fertility-sparing treatment is particularly important for these patients [3].

Over recent years, research studies have increasingly recognized the safety and efficacy of a conservative form of treatment involving high-dose progestin for patients with AEH and early stage endometrioid adenocarcinoma (EEC) [4–10]. Fertility-sparing therapy aims to temporarily reverse endometrial lesions via the use of a large amount of progesterone, thus creating a safe time window for pregnancy and fertility. It is highly evident that both clinicians and patients are now interested in both oncological and pregnancy outcomes. It is now recommended that individuals with a desire to have children should aim to become pregnant immediately after achieving CR of the disease. Relevant studies have already reported the pregnancy outcomes of patients with EEC and AEH patients following such therapeutic intervention [11–17].

In patients with endometrial disease and receiving conservative treatment, there are several factors that might interfere with the outcomes of pregnancy; including the initial pathological changes in the endometrium; the high concentrations of progesterone during treatment; repetitive intrauterine operations, such as diagnostic dilatation and curettage biopsy (D&C) with or without hysteroscopy, for the evaluation of other conditions, including disease progress, relapse, and intrauterine adhesion (IA). Young patients are usually complicated by polycystic ovarian syndrome (PCOS), obesity, and other metabolic diseases; these can change endometrial receptivity and make it difficult to maintain pregnancy. These factors associated with pregnancy after hormonal treatment have yet to be investigated in detail. This study aimed to investigate pregnancy outcomes and analyze factors associated with pregnancy in young EEC and AEH patients who received fertility-sparing management. We also investigated the effects of pregnancy on recurrence.

## Methods

### Study design and patients

This was a retrospective study involving patients receiving fertility-sparing treatment in Peking University People’s Hospital in China between September 2011 and December 2019. This research was approved by our Institutional Review Board (Approval number: 2020PHB063-01). Medical records were used to collate data for each patient relating to clinical characteristics, treatment protocols, and oncological and pregnancy outcomes. The STROBE guidelines were used to facilitate this observational study [18].

Patients were selected for fertility-sparing treatment if they fulfilled the following conditions: (1) age ≤ 45 years with a strong desire for fertility; (2) diagnosed with AEH or endometrioid adenocarcinoma (grade 1 or grade 2); (3) lesions confined to the endometrium, as determined by imaging (MRI); (4) positive expression of estrogen receptor and progesterone receptor expression; and (5) the patient signed an informed consent form and underwent an appropriate period of follow-up. Patients were excluded if there was a contraindication for progestin treatment and fertility, or if they had severe medical complications or other malignant diseases of the reproductive system. EEC/AEH patients were considered to be eligible for this study if they had undergone fertility-sparing therapy, had achieved CR of endometrial lesions, and had a strong desire to conceive. Pathological diagnosis was confirmed by experienced gynecological pathologists in accordance with World Health Organization guidelines.

### Treatments and follow-up

Patients receiving fertility-sparing therapy were administered with high-dose progestin medications including medroxyprogesterone (MPA), megestrol acetate (MA), gonadotropin-releasing hormone agonist (GnRH-a), with or without additional metformin. Patient response was evaluated by endometrial sampling (performed with hysteroscopy) at three-month intervals. Patients with pathological CR were allowed to terminate the treatment protocol and were recommended for a pregnancy attempt, which was called ‘pregnancy permission’ in the following. Simultaneous maintenance therapy, including low-dose progestin or the levonorgestrel intrauterine system (LNG-IUD), was given to prevent relapse. The follow-up was scheduled every 3–6 months for a general gynecological examination and transvaginal ultrasound, and the biopsy of endometrium was held every six months until they got pregnant. Women failing to conceive spontaneously, or those with a history of infertility, were offered ART treatments, including ovulation induction with or without intrauterine insemination (IUI), in vitro fertilization, and embryo transfer (IVF-ET). In order to minimize the risk of tumor recurrence, we applied letrozole alone or combined with gonadotropin, for ovarian stimulation. All women were followed up with regards to pregnancy status and cancer recurrence.

### Outcome measures

In this current study, subjects were assigned into two groups (a pregnancy group and a non-pregnancy group), based on pregnancy outcome. The primary outcome was influencing factors associated with pregnancy success. The secondary outcome was the effects of pregnancy on disease recurrence. ‘Time to CR’ was used to define the period from the initiation of treatment to the first pathologically confirmed CR of lesions. After the CR had been achieved, some doctors preferred to protract the medication time for up to 3–12 months in order to achieve greater levels of lesion inhibition; this was referred to as ‘prolonged therapy’. The ‘age and BMI of pregnancy permission’ was defined as the age and BMI at the time that a pregnancy trial was allowed. The thickest endometrium in the proliferative phase after the withdrawal of treatment was measured by transvaginal ultrasound and referred to as ‘endometrial thickness after CR’. The time duration from the permitted pregnancy trial time to the last menstruation period of successful pregnancy was defined as ‘pregnancy interval (PI)’. Time to relapse was defined as the duration from the termination of prior treatment to the time at which recurrence was pathologically confirmed; this was used to calculate RFS. The longest observation time was set to be 5 years.

### Statistical analysis

Baseline characteristics were tabulated for descriptive statistics and categorical variables were compared using the Chi-squared test; continuous variables were compared using the Student’s t-test. A Cox proportional hazard regression model was used for univariate and multivariate analysis to identify factors associated with pregnancy success; these factors were determined with a hazard ratio (HR) and 95% confidence interval (CI). The model included each covariate individually and covariates were included as potential confounders in the final models if they changed the estimates of factors affecting pregnancy by more than 10% or were significantly associated with clinical pregnancy outcomes. The Kaplan–Meier method was used to calculate a patient’s probability of pregnancy and the RFS and curves were tested for statistical significance using the log-rank test for univariable analysis. *P* < 0.05 was considered to be statistically significant. Data were analyzed by the R statistical package (The R Foundation; http://www.r-project.org; version 3.6.3) and Empower (R) software (www.empowerstats.com, X&Y solutions, inc. Boston, Massachusetts).

## Results

### Patient characteristics

A total of 68 patients with EEC or AEH were treated with fertility-sparing therapy, achieved CR, and attempted to conceive after hormonal treatment. Patient characteristics for the pregnancy group and the non-pregnancy group are summarized in Table 1. No significant difference was found in terms of the initial treatment age and BMI, parity, internal diseases, myometrium invasion as determined by MRI, histological type, treatment protocols, metformin use, maintenance therapy, and conception methods, when considered between the two groups of patients.

**Table 1:** Demographics and clinicopathological characteristics of EEC/AEH patients after achieving CR and attempting to conceive

	Total	Non-pregnant	Pregnant	*P* value
Patients (n)	68	32	36	-
Age of initial treatment (years)	30.4 ± 3.9	31.0 ± 3.7	29.9 ± 4.1	0.272 ^a^
Age of pregnancy permission (years)	31.9 ± 4.2	32.6 ± 3.8	31.2 ± 4.5	0.190 ^a^
BMI ^c^ of initial treatment (kg/m^2^)	25.9 ± 4.2	26.9 ± 4.2	25.0 ± 3.9	0.051 ^a^
BMI of pregnancy permission (kg/m2)	25.9 ± 3.8	27.2 ± 3.7	24.8 ± 3.6	0.007
Irregular menstrual cycle (%)	35 (51.5)	18 (56.2)	17 (47.2)	0.457 ^b^
Infertility (%)	27 (39.7)	14 (43.8)	13 (36.1)	0.520^b^
Nulliparity (%)	62 (91.2)	30 (93.8)	32 (88.9)	0.481 ^b^
Diabetes mellitus type 2 (%)	11 (16.2)	3 (9.4)	8 (22.2)	0.151 ^b^
Insulin resistance (%)	21 (30.9)	10 (31.3)	11 (30.6)	0.950 ^b^
High blood pressure (%)	6 (8.8)	2 (6.2)	4 (11.1)	0.481 ^b^
Thyroid diseases (%)	9 (13.2)	4 (12.5)	5 (13.9)	0.866 ^b^
Myometrium invasion in MRI (%)	16 (33.3)	8 (34.8)	8 (32.0)	0.581 ^b^
PCO on ultrasonography (%)	37 (54.4)	20 (62.5)	17 (47.2)	0.207 ^b^
PCOS^d^	27 (39.7)	13 (40.6)	14 (38.9)	0.884^b^
CA125 (U/mL)	22.4 ± 22.6	22.7 ± 16.2	22.2 ± 27.2	0.926 ^a^
Histological type				0.214 ^b^
AEH	39	16	23	
EEC G1	21	10	11	
EEC G2	8	6	2	
Treatment protocol (%)				0.831 ^b^
MPA 250 mg, once daily	45 (67.2)	21 (65.6)	24 (68.6)	
MPA 500 mg, once daily	8 (11.9)	5 (15.6)	3 (8.6)	
MA 160-320 mg, once daily	9 (13.4)	4 (12.5)	5 (14.3)	
GnRH-a	5 (7.5)	2 (6.2)	3 (8.6)	
Adjuvant metformin (%)	25 (36.8)	15 (46.9)	10 (27.8)	0.103 ^b^
Maintenance therapy (%)				0.223 ^b^
None	11 (16.2)	6 (18.8)	5 (13.9)	
Progestin	45 (66.2)	18 (56.2)	27 (75.0)	
LNG-IUD	12 (17.6)	8 (25.0)	4 (11.1)	
Conception method (%)				0.246 ^b^
Natural	19 (27.9)	12 (37.5)	7 (19.4)	
Ovulation induction ± IUI	9 (13.2)	4 (12.5)	5 (13.9)	
IVF-ET	40 (58.8)	16 (50.0)	24 (66.7)	

*AEH* atypical endometrial hyperplasia, *BMI* body mass index, *CA* cancer antigen, *EEC* early stage endometrial cancer, *GnRH-a* gonadotropin-releasing hormone agonist, *IUI* intrauterine insemination, *IVF-ET* in vitro fertilization and embryo transfer, *LNG-IUD* levonorgestrel intrauterine system, *MA* megestrol acetate, *MPA* medroxyprogesterone acetate, *PCO* polycystic ovary

^a^Pregnancy versus Non-pregnancy (Student’s t test)

^b^Pregnancy versus Non-pregnancy (Chi-squared test)

^c^BMI, kg/m2 (Chinese Society for the Study of Obesity: normal BMI 18.5–23.9; overweight 24–28; obesity > 28)

^d^Diagnostic criteria: the Rotterdam criteria 2003

### Pregnancy outcomes after fertility-sparing treatment

**Fig. 1:**
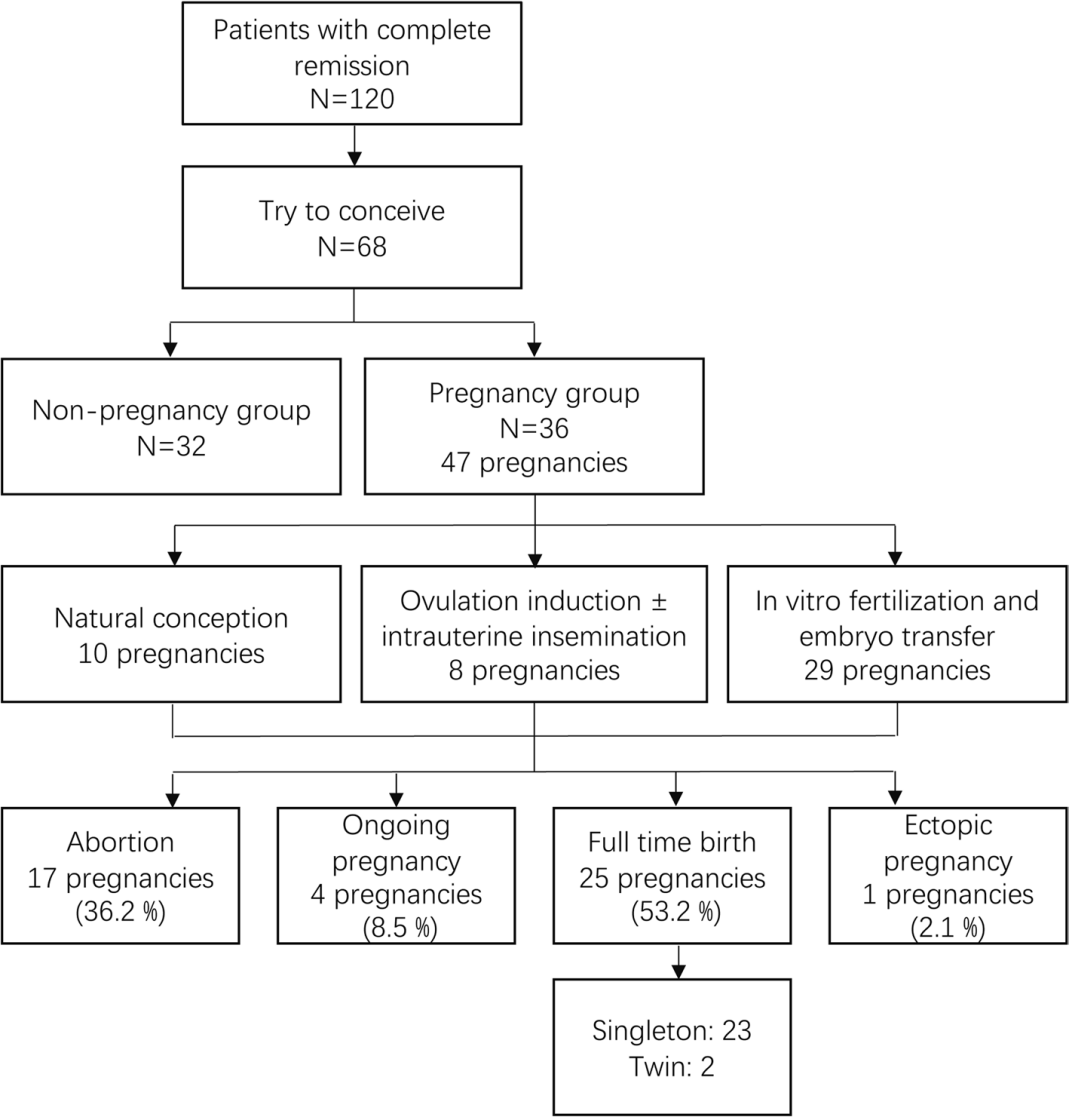
Pregnancy outcomes of patients with early stage endometrial cancer or atypical endometrial hyperplasia after achieving complete remission

A total of 36 patients became pregnant with 47 pregnancies (Fig. 1). Ten pregnancies were achieved by NC, five by ovulation stimulation, three by ovulation stimulation with IUI, and 29 by IVF-ET. The outcomes of the 47 pregnancies were as follows: 17 abortions (36.2%), 4 ongoing pregnancies (8.5%), 25 live births (53.2%), and 1 ectopic pregnancy (2.1%). Four patients (11.1%) experienced recurrent pregnancy loss (RPL) (two miscarriages each). The total number of live births was 27, including two twins. Two EEC patients successfully delivered twice following CR of the disease. Four cases experienced incompetent internal os of the cervix. Cesarean section was performed in ten cases and vaginal delivery occurred in fifteen cases. With respect to the non-pregnancy group, 20 (62.5%) of the patients received ART treatment and 16 (50%) of them underwent IVF-ET but failed to conceive. In addition, there were three patients undergoing hysterectomies after multiple recurrences and the others continued the conservative treatment and then received maintenance therapy. While there were two patients choosing surgery in the pregnant group, one after the spontaneous abortion and recurrence, and the other underwent hysterectomy at the same time of full-term cesarean section.

### Factors related to pregnancy outcomes

As shown in Table 2, the time required for lesions to disappear was considerably shorter in the pregnancy group (4.2 ± 2.5 months) than in the non-pregnancy group (6.4 ± 4.0 months), as was the relapse before pregnancy (16.7% *vs.* 40.6%). The BMI at the time of pregnancy permission was significantly lower (24.8 ± 3.6 years *vs.* 27.2 ± 3.7 years) and significantly fewer hysteroscopy (HS) procedures were performed (3.4 ± 1.0 *vs.* 4.1 ± 1.2) in the pregnancy group than in the non-pregnancy group. Endometrial thickness after CR was higher in the pregnancy group (0.7 ± 0.2 cm) than in the non-pregnancy group (0.6 ± 0.2 cm). Age at the time of pregnancy permission, IA status, and prolonged treatment time, were not statistically significant when compared between the two groups.

**Table 2:** Univariate and multivariate Cox regression model of variables associated with pregnancy outcomes in AEH/EEC patients after fertility-sparing treatment

Variables	Non-pregnant	Pregnant	HR (95% CI) *P* value		
	*N* = 32	*N* = 36	Crude model	Adjusted model I	Adjusted model II
Age of pregnancy permission (years)	32.6 ± 3.8	31.2 ± 4.5	1.0 (0.9, 1.1) 0.717	1.0 (1.0, 1.1) 0.502	1.0 (0.9, 1.1) 0.554
BMI of pregnancy permission (kg/m^2^)	27.2 ± 3.7	24.8 ± 3.6	0.9 (0.8, 1.0) 0.061	0.9 (0.8, 1.0) 0.045	0.9 (0.8, 1.0) 0.031
< 24	3 (9.4%)	18 (50.0%)	Refrence	Refrence	Refrence
≥ 24	29 (90.6%)	18 (50.0%)	0.4 (0.2, 0.8) 0.012	0.4 (0.2, 0.8) 0.010	0.4 (0.2, 0.8) 0.010
Additional prolonged therapy (months)	2.1 ± 2.6	2.2 ± 1.9	1.0 (0.9, 1.2) 0.794	1.0 (0.9, 1.2) 0.705	1.0 (0.8, 1.2) 0.770
0	17 (53.1%)	13 (36.1%)	Refrence	Refrence	Refrence
3	9 (28.1%)	19 (52.8%)	2.1 (1.0, 4.2) 0.044	2.9 (1.2, 7.0) 0.018	3.0 (1.1, 8.3) 0.037
≥ 6	6 (18.8%)	4 (11.1%)	0.9 (0.3, 2.7) 0.829	1.2 (0.3, 4.0) 0.817	0.7 (0.2, 2.7) 0.603
Time to CR (months)	6.4 ± 4.0	4.2 ± 2.5	0.9 (0.7, 1.0) 0.049	0.9 (0.7, 1.0) 0.109	0.8 (0.7, 1.0) 0.032
< 6	12 (37.5%)	28 (77.8%)	Refrence	Refrence	Refrence
≥ 6	20 (62.5%)	8 (22.2%)	0.3 (0.1, 0.7) 0.004	0.3 (0.1, 0.8) 0.014	0.2 (0.1, 0.6) 0.004
Intrauterine adhesion					
No	16 (50.0%)	26 (72.2%)	Refrence	Refrence	Refrence
Yes	16 (50.0%)	10 (27.8%)	0.5 (0.2, 1.1) 0.082	0.6 (0.3, 1.4) 0.244	0.6 (0.3, 1.4) 0.272
Number of HS	4.1 ± 1.2	3.4 ± 1.0	0.7 (0.5, 0.9) 0.007	0.7 (0.5, 1.0) 0.024	0.6 (0.4, 0.8) 0.004
Endometrial thickness after CR (cm)	0.6 ± 0.2	0.7 ± 0.2	5.0 (0.9, 28.8) 0.072	7.3 (0.9, 57.3) 0.058	8.8 (1.1, 73.0) 0.043
Relapse before pregnancy					
No	19 (59.4%)	30 (83.3%)	Refrence	Refrence	Refrence
Yes	13 (40.6%)	6 (16.7%)	0.3 (0.1, 0.7) 0.006	0.2 (0.1, 0.6) 0.004	0.2 (0.1, 0.5) 0.001

Crude model adjust for: None

Adjust model I adjust for: Histological type; Treatment protocol

Adjust model II adjust for: Histological type; Treatment protocol; HOMA; PCO on ultrasonography; Parity

BMI, kg/m^2^ (Chinese Society for the Study of Obesity: normal BMI 18.5–23.9; overweight 24–28; obesity > 28)

*BMI* body mass index, *CR* complete remission, *CI* confidence interval, *HR* hazard ratio, *HS* hysteroscopy, *PCO* polycystic ovary

**Fig. 2:**
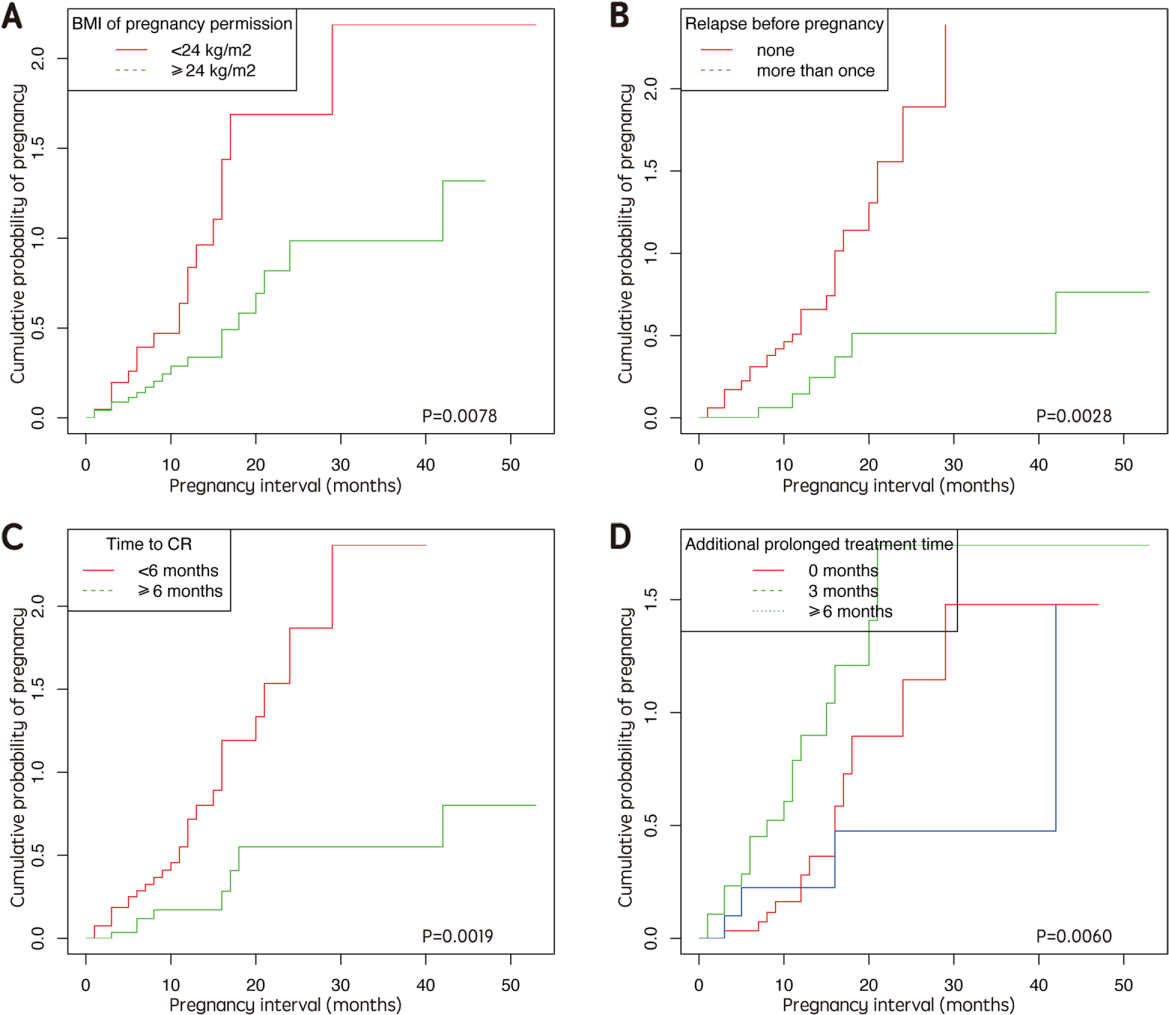
Cumulative probability of pregnancy curves in EEC/AEH patients after achieving CR and attempting to conceive. **A** The cumulative probability of pregnancy in patients with BMI < 24 kg/m^2^ and BMI ≥ 24 kg/m^2^. Overweight and obese patients had a lower probability of pregnancy. **B** The cumulative probability of pregnancy in patients never relapsed before pregnancy and relapsed for once or more. No recurrence of disease related to a higher probability of pregnancy. **C** The cumulative probability of pregnancy in patients with cure time to CR shorter than 6 months and long than or equal to 6 months. The latter showed a lower probability of pregnancy. **D** The cumulative probability of pregnancy in patients with different additional prolonged treatment time. Patients receiving an additional 3 months of treatment got a higher probability of pregnancy than those with no prolonged treatment or 6 months and even longer prolonged treatment. BMI, kg/m^2^ (Chinese Society for the Study of Obesity: normal BMI 18.5–23.9; overweight 24–28; obesity > 28). AEH: atypical endometrial hyperplasia; BMI: body mass index; CR: complete remission; EEC: early stage endometrial cancer

**Fig. 3:**
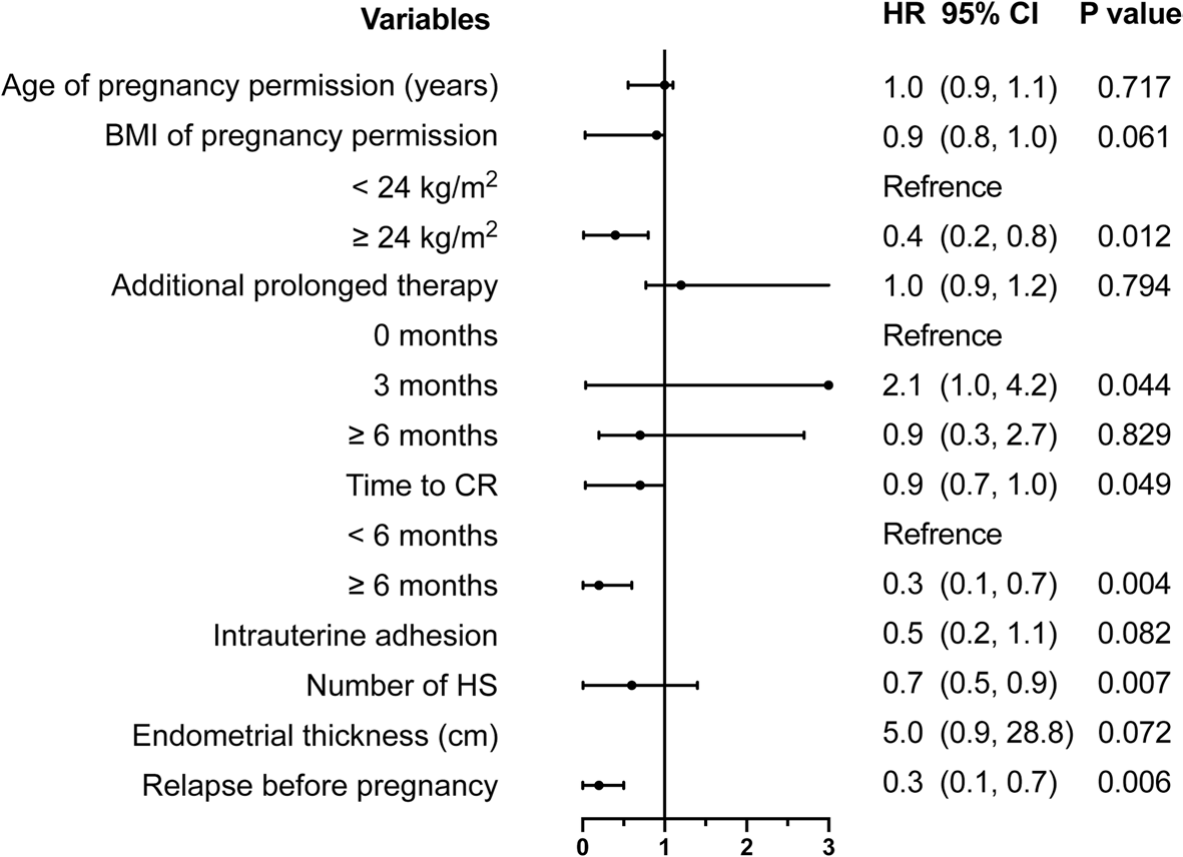
Adjusted Cox regression model of variables associated with pregnancy outcomes in AEH/EEC patients after achieving CR. Adjusted factors: parity, histological type, treatment protocol, HOMA, PCO on ultrasonography. BMI: body mass index; CR: complete remission; CI: confidence interval; HR: hazard ratio; HS: hysteroscopy; PCO: polycystic ovary

Univariate analysis revealed significant differences between the two groups in terms of BMI at pregnancy permission, time to CR, the number of HS procedures, and relapse before pregnancy (Table 2). Stratified log-rank tests of BMI at the time of pregnancy permission, the time to CR, additional prolonged treatment time, and relapse status (Fig. 2) all resulted in differences in the cumulative probability of pregnancy. Table 2 shows multivariate Cox regression analysis; the final adjusted model revealed that several factors were negatively correlated with a successful pregnancy, including higher BMI, longer time to CR, a greater number of HS procedures, a thinner endometrium after CR, the incidence of IA, and relapse prior to pregnancy. Subgroup analysis revealed that overweight and obese women were 60% less likely to become pregnant than those who were not overweight or obese (Fig. 3).

### The effects of pregnancy on recurrence

**Fig. 4:**
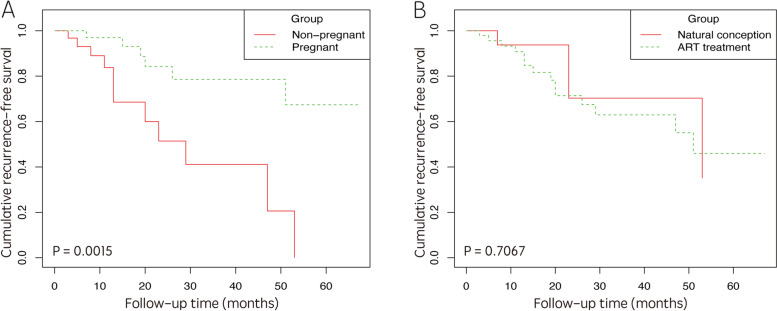
Cumulative RFS curves in fertility-sparing EEC/AEH patients after the successful or failed pregnancy. **A** The cumulative RFS in patients of pregnancy group and non-pregnancy group. With successful pregnancy, patients had longer RFS than failed pregnancy group patients. **B** The cumulative RFS in patients with ART treatment and natural conception. There was no difference in RFS between the two groups. AEH: atypical endometrial hyperplasia; ART: assisted reproductive technology; EEC: early stage endometrial cancer; RFS: recurrence-free survival

The recurrence rate was 16.7% (6/36) and 40.6% (13/32) in the pregnancy and non-pregnancy group, respectively. For patients in the pregnancy and non-pregnancy group, the mean RFS of the patients who achieved pregnancy and those who did not were 27.6 months (range: 2–67 months) and 14.8 months (range: 1–53 months), respectively (*P* = 0.002). In addition, there were five cases undergoing a total of seven recurrences after abortion in pregnant group. There was no significant difference with regards to cumulative RFS when compared between cases with ART treatment and those with NC (*P* = 0.707) (Fig. 4).

## Discussion

In this retrospective study, young EEC/AEH patients receiving fertility-sparing treatment with high-dose progestin were divided into pregnancy and non-pregnancy groups. These groups were then compared so that we could investigate treatment factors that might be associated with pregnancy after achieving CR. In the pregnancy group, 78.7% of pregnancies were achieved by ART treatment; 61.7% of these were achieved by IVF-ET. The live birth rate for the cases produced by ART was 53.2%; this is an excellent indicator of successful fertility-sparing management. The goal of such management is to obtain a baby, not just to get pregnant.

Until now, there has been inadequate evidence relating to the indicators for successful pregnancy after fertility-sparing treatment in patients with EEC/AEH. Osamu et al.identified several factors that were related to pregnancy, including recurrence before conception, endometrial thickness during ovulation, and patient age at the time that pregnancy was attempted [12]. Chae et al.concluded that tumor grade is a crucial factor influencing pregnancy outcomes [13]. Considering the different protocols and the length of time taken to get pregnant, and that these factors can be influenced by menstrual cycle, the work environment, and even epidemics, we calculated the interval from pregnancy permission to achieving pregnancy, or the termination of pregnancy, or recurrence, as ‘follow-up’ time. This allowed us to conduct a Cox regression analysis; this was important because such analysis has not been applied in other studies.

In general, advanced age is a recognized risk factor for female infertility, although we found that the age at the time of pregnancy permission had little impact on pregnancy success in our study. This is likely to be due to the fact that most of the patients included in our study were young; the mean age of females in the two groups was 31.2 ± 4.5 years and 32.6 ± 3.8 years, respectively, when they were allowed to conceive. On the other hand, due to the small number of cases included in this study, the association between age-related factors and pregnancy needs to be confirmed with more centers and larger samples of research.

Previous studies have reported reduced levels of fertility in obese women [19]. Our study demonstrated that a higher BMI was associated with a lower probability of conception. From our experience, it is more arduous for obese patients to get CR. These patients are more likely to relapse. Collectively, these factors result in a reduced chance of pregnancy. The underlying reason for this is most likely due to the surplus of endogenous estrogens produced by body fat and the fact that this can resist progestin therapy [8]. Furthermore, obesity could contribute to ovulatory dysfunction in women, thus making it difficult for women to conceive, either by NC or ART [15]. It is therefore essential to educate patients and instill the concept of weight loss by improving lifestyle and dietary structure.

The appropriate time window for evaluating the initial response to progestin remains unclear, although most studies prefer to focus on the three months following treatment. Negative endometrial findings have been reported after 10 weeks of treatment with MPA [20]. Koskas et al. demonstrates that the CR rate after 3, 6, 12, 18, and 24 months of treatment, were 30.4%, 72.4%, 78.0%, 80%, and 81.4%, respectively [6]. Our present findings indicate that the cure time to CR is clearly associated with the probability of pregnancy. From this standpoint, it is reasonable to suggest if remission occurs quickly, then the condition was not so severe, and thus, the chances of becoming pregnant are higher.

We should also consider the length of the fertility-sparing protocol. Some researchers have proposed that for the sake of higher efficacy, the appropriate duration of progesterone treatment should not be less than one year [9]. Niwa et al. stated that the drug should be administered for at least 6 months or 2 months after a lesion disappears [10]. Some Chinese researchers have suggested that a better form of management would be to continue therapy for 3–6 months after CR, even if the lesions disappeared after three months of therapy. It appears that clinicians hold various points of view with regards to the course of treatment. In our series of patients, 44.1% (30/68) of patients terminated their hormonal therapy when they first achieved CR, 41.2% (28/68) of patients received additional treatments for another three months, while 14.7% (10/68) of patients continued treatment for 6 months or longer. It is feasible that long-term high-dose progesterone might affect endometrial receptivity and reduce the pregnancy rate of early assisted pregnancies. In the future, it will be necessary to conduct randomized trials to confirm this issue.

Our analysis revealed significant differences in the number of HS procedures between the two groups (*P* = 0.011). We also found that the mean endometrial thickness after treatment in the pregnancy group was thicker than that in the non-pregnancy group. During treatment, frequent uterine cavity surgery, particularly D&C, is likely to cause mechanical damage to the endometrium. This may result in endometritis and a thinner endometrium and impair endometrial receptivity during fertility-sparing treatment [21]. These findings were similar to those published by Elizur et al. [22] and Fujimoto et al. [23]. Furthermore, repeated intrauterine operations might increase the risk of IA, and the IA rate in the whole cohort, 50% in non-pregnancy group and 27.8% in pregnancy group, separately) was quite high in our study. This probably related to the close monitoring with endometrial sampling (biopsies or D&C) through frequent uterine cavity operations, increasing the risk of IA and endometrium damage in this cohort. On the other hand, most of them we observed were mild, film-like adhesions, and it might be due to the improvement of detection and diagnosis rate of IA under hysteroscopy. Additionally, the RPL rate (11.1%) was relatively high in this cohort, which was an interesting finding and might be tied to the increased presence of IA and endometrium damage.

The recurrence of lesions has become a significant problem that we cannot ignore following hormonal therapy. The recurrence rate published ranged from 35% to 62.2% [12, 24, 25]. In our series, tumor relapse occurred in 16.7% (6/36) and 40.6% (13/42) of patients in the pregnancy and non-pregnancy group, respectively. We also found that patients experiencing relapse prior to pregnancy were 80% less likely to conceive than those without recurrence, thus implying that recurrence is highly detrimental to the establishment of a successful pregnancy. Maintenance treatment with low-dose cyclic progestin, or a progestin-containing IUD, is known to be associated with a lower risk of recurrence [8]. It is important that physicians should take active measures, such as standardized treatment protocols combined with reasonable maintenance treatment, and implement ART treatment as early as possible.

According to our analysis, pregnancy exhibits a positive effect on the endometrium. We found that the time to recurrence was longer in the pregnant group than that in the non-pregnant group (*P* = 0.002), and the recurrence rate was twice as high in the pregnancy group than the non-pregnancy group. These findings are similar to those reported by Park et al., who stated that the relapse rate was 20.5% and 36.6% in pregnant and non-pregnant groups. The multivariate analysis also revealed a significant improvement in RFS in the pregnant group [11]; this was identical to the findings published by Chae et al. [13]. High levels of hormones during pregnancy do not promote the progression of endometrial lesions, but do provide the same effects as a highly effective progesterone treatment. During delivery and the puerperal process, the decidual endometrium is completely exfoliated; this is equivalent to curettage and plays a therapeutic effect on endometrial lesions to prevent relapse, at least to some extent [8, 26]. On the other hand, pregnancy stops the vicious cycle of estrogen exposure caused by PCOS in obese females. However, regular tumor follow-up should be continued during pregnancy, with a follow-up interval of 6 months [27].

Usually, the use of fertility drugs lead to an elevation of estrogen during the ovulation induction cycle; this probably increases the risk of EC progression or recurrence. Azim and Oktay described the use of letrozole in conjunction with gonadotropins for controlled ovarian stimulation in order to avoid high estrogen levels associated with conventional regimens [28]. This protocol is widely applied by our unit; In our present series, the use of ART did not result in an increased recurrence of EC and therefore did not compromise the RFS of our patients, which were consistent with the results published in Chao et al. and Ichinose et al. [16, 17]. Actually, the RFS of our patients achieving pregnancy was better regardless of ART treatment. Previous research has shown that the probability of recurrence is higher when the time taken to achieve CR is longer [6]. Considering that many AEH/EEC patients are infertile, it is important to apply ART soon after CR is achieved.

This study had some limitations that need to be considered. First, this study was carried out in a single center with a relatively small number of patients. Second, our main methodology was retrospective chart review; it is therefore possible that selection bias could have occurred. Third, the details of IVF treatment are not fully collected, and we are going to present cases involving outcomes of IVF cycles and detailed IVF treatment information in the following study. Furthermore, a variety of confounding factors could have been present in our analysis, including performance status and patient/physician bias relating to treatment or pregnancy choices. The outcomes of the present study should be interpreted with caution and confirmed by large-scale research in the future. However, our study benefited greatly from the fact that it incorporated a long follow-up period and took into account pregnancy intervals. Ultimately, our study was more likely to represent a real-world circumstance of a patient attempting to conceive and perhaps was more generalizable than what might be expected from a prospective trial. Finally, we must continue to seek strategies that effectively benefit patients with conception after fertility-sparing therapy while safeguarding patient priorities, whenever safe and feasible.

## Conclusions

In conclusion, we identified several factors that were positively associated with a successful pregnancy, including a normal BMI at the time of pregnancy permission, a shorter cure time to CR, and a fewer number of HS procedures. A thinner endometrium, and relapse prior to pregnancy, may have a negative effect on pregnancy. Moreover, successful pregnancy might provide protection to the endometrium and reduce the recurrence. The application of ART did not increase the risk of recurrence. A prospective study with a large cohort is now indispensable to confirm our findings.

## Data Availability

The datasets used and/or analysed during the current study are available from the corresponding author on reasonable request.

## Abbreviations

RFS: Recurrence-free survival
CR: Complete remission
ART: Assisted reproductive technology
NC: Natural conception
EC: Endometrial cancer
AEH: Atypical endometrial hyperplasia
EEC: Early stage endometrioid adenocarcinoma
D&C: Dilatation and curettage biopsy
PCOS: Polycystic ovarian syndrome
IA: Intrauterine adhesion
MPA: Medroxyprogesterone
MA: Megestrol acetate
GnRH-a: Gonadotropin-releasing hormone agonist
LNG-IUD: Levonorgestrel intrauterine system
IUI: Intrauterine insemination
IVF-ET: In vitro fertilization and embryo transfer
PI: Pregnancy interval
HR: Hazard ratio
CI: Confidence interval
HS: Hysteroscopy
